# Support of Tumor Endothelial Cells by Chemokine Receptors

**DOI:** 10.3389/fimmu.2019.00147

**Published:** 2019-02-08

**Authors:** Nicole Salazar, Brian A. Zabel

**Affiliations:** ^1^Department of Biology, San Francisco State University, San Francisco, CA, United States; ^2^Palo Alto Veterans Institute for Research, Veterans Affairs Palo Alto Health Care System, Palo Alto, CA, United States

**Keywords:** chemokine receptor, chemoattractant, endothelial cell, tumor vasculature, tumor microenvironment

## Abstract

Tumor-associated vascular endothelium comprises a specialized and diverse group of endothelial cells that, although not cancer themselves, are integral to cancer progression. Targeting the tumor vasculature can have significant efficacy in reducing tumor burden, although loss of efficacy due to acquisition of resistance mechanisms is common. Here we review mechanisms by which tumor endothelial cells (TEC) utilize chemokine receptors to support tumor progression. We illustrate how chemokine receptors support and may serve as functional markers of the diverse TEC population. We focus on ACKR1 (DARC), ACKR3 (CXCR7), CXCR4, and CCR2, as these are the best studied chemokine receptors in TEC; and suggest that targeting these receptors on the tumor vasculature may prove efficacious in slowing or reversing tumor growth. We also mention CXCR2 and CXCR3 as important mediators or tumor angiogenesis, given their distinct roles with angiogenic and angiostatic chemokines, respectively.

## Introduction

The endothelium consists of a network of endothelial cells (ECs) that form the inner lining of blood and lymphatic vessels. Endothelium is present only in vertebrates, including the hagfish, the oldest of extant vertebrates (1, 2). The endothelium is critical for the trafficking of leukocytes between the vasculature and the underlying tissues. The endothelium is also involved in most major pathologic conditions, from cancer, to cardiovascular disease, neuroinflammation, diabetes, and high blood pressure, either as a primary determinant of pathophysiology or as a victim of collateral damage(3).

In the context of cancer, endothelial cells form the inner lining of the blood vessels that make up part of a growing tumor. Compared to normal endothelial cells, tumor endothelial cells (TEC) become morphologically and phenotypically dysregulated at the cellular and molecular level, much like the tumor itself. TEC retain their ontological endothelial identity, remain distinct from cancer cells, and are not immortal. TEC support is, however, a major component of the tumor microenvironment, not only irrigating the tumor with nutrients, but also affecting the immune cell infiltrate and stromal composition of the tumor (3, 4). The dysregulation of ECs within the tumor leads to loss of proper vascular barrier function and gain of properties that provide tumors with survival advantages.

**Figure 1:**
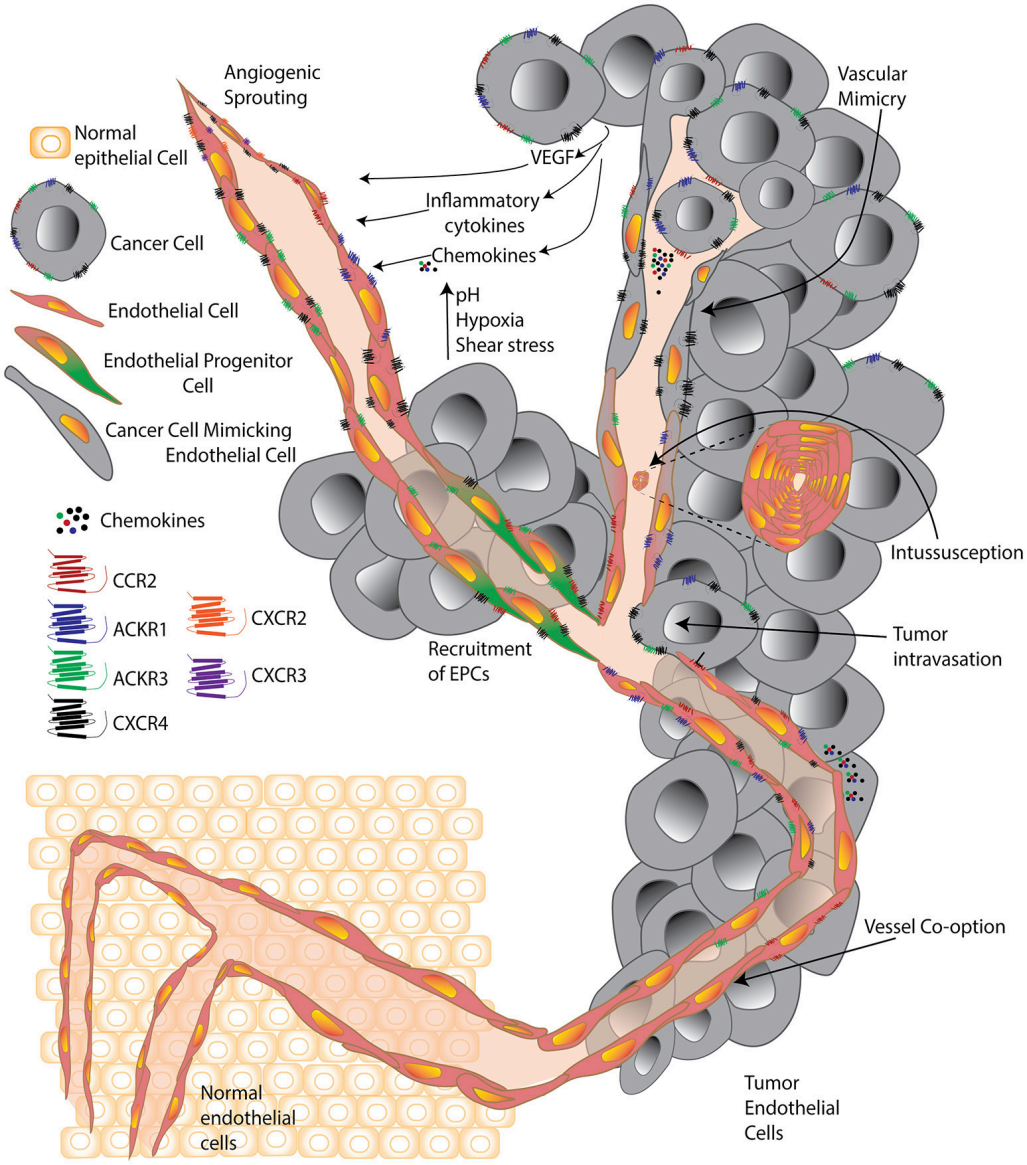
A cell is known by the company it keeps. Simplified schematic comparing tumor endothelial cells surrounded by normal healthy cells and surrounded by cancer cells. Normal endothelial cells are significantly altered within the tumor environment and forced by tumors to undergo processes including classic angiogenic sprouting, vasculogenesis, intussusception, vessel co-option, vascular mimicry, and progenitor differentiation. Within the tumor, environmental factors including growth factors, cytokines, chemokines, high acidity, low oxygen, and high pressure, contribute to push individual tumor endothelial cells to acquire unique features and a vast diversity that supports the progression of the expanding tumor microenvironment.

Misconceptions regarding the origin of TEC have been clarified in the last few years with the advent of improved technologies that have enabled a detailed compositional analysis of these cells and uncovered a vast repertoire of functional activities and associated markers. Key misconceptions included that (i) tumor endothelial cells were derived from the tumor, (ii) TECs were similar to their normal counterparts; and (iii) targeting only vascular endothelial growth factor (VEGF) would be sufficient to destroy the tumor they were a part of. Interestingly, the heterogeneity of TECs is such that it is extremely challenging to pinpoint a single overall function or modus operandi that defines the TEC population. Tumors hijack otherwise normal homeostatic or developmental vascular endothelial processes, such as angiogenic sprouting or vasculogenesis; or engage in vessel co-option or other mechanisms, such as intussusception (splitting of pre-existing vessels to give rise to daughter vessels), and parasitize the host's vascular system to promote tumor survival, growth, and dissemination (5–8) (Figure 1).

Tumor angiogenesis results from growth factor and chemokine-dependent EC proliferation. Classic EC proliferation is stimulated by VEGFs (Table 1). Depending on the angiogenic cytokine, distinct characteristics evolve: for example, VEGF-B and placental growth factor (PlGF) bind and activate VEGF-R1, which is responsible for hematopoiesis, monocyte migration, EC metabolism, and arteriogenesis (9). VEGF-A and VEGF-C bind and activate VEGF-R2, which initiates and sustains the classic angiogenesis process, giving rise to blood endothelial vessels (BECs) (10–12). VEGF-C and VEGF-D bind and activate VEGF-R3, which gives rise to lymphatic endothelial cells (LEC). TEC derived from angiogenic processes are identifiable within the tumor as early as 3 days after tumor inoculation in preclinical models (13).

**Table 1:** Classic endothelial cell type determined by Vascular Endothelial Growth Factor (VEGF) type.

VEGFR-1	VEGF-B, PlGF	Arterial ECs
VEGFR-2	VEGF-A	MV, BECs
VEGFR-3	VEGF-C, VEGF-D	LECs

*Classical, normal endothelial cell proliferation is stimulated by VEGFs. Depending on the angiogenic cytokine, distinct characteristics evolve for each endothelial cell type*.

Given the conflicting evidence on whether or not there exists bone marrow-derived precursors that are bona fide endothelial progenitors cells (EPC), we adopt a more general definition of vasculogenesis as the process of new blood vessel formation assisted by pro-angiogenic bone marrow-derived precursors, if not actual EPC (14). Tumors utilize vasculogenic mechanisms to form new blood vessels (15, 16). EPCs are a subtype of stem-like cells with high proliferative potential that mobilize from the bone marrow and home to tumor sites in response to tumor-secreted cytokines/chemokines, where they continue the cycle to mature into ECs and secrete proangiogenic factors to facilitate vascularization of tumors (17). The main participants are VEGF-A/VEGFR-2, CXCL12/CXCR4/ACKR3, CXCL8/CXCR1, CXCL1,2,3,5,6,7,8/CXCR2, CCL2/CCR2, and CCL5/CCR5 (15, 17–19). An important driver of chemokine gradients in the tumor microenvironment are cancer cells under aerobic glycolysis, which produce lactic acid that activates NF-κB and induces CXCL8 expression in vascular endothelial cells, resulting in angiogenesis in breast and colon cancer (20, 21). CXCL8 also upregulates CXCR7/ACKR3, which is involved in stemness features of cancer cells suggesting it is also likely involved in EPC mobilization (22–24).

Vascular co-option is a process by which tumor cells surround host vessels and incorporate host–tissue capillaries within the tumor, thereby eliminating the need for new vessel formation. Vessel co-option occurs mainly in highly vascularized tissues, such as liver, lungs, and brain. Ronca et al. (16) explain that these tumors are considered non-angiogenic and are *less* likely to respond to antiangiogenic therapy. Ronca et al. (16) make the point that a critical limitation of studies is that most tumor endothelial cells are studied using known markers of ECs such as CD31, CD34, and/or vWF. However, these markers also stain co-opted host vessels. Smooth muscle cell actin (SMA), which stains pericytes that cover mature vessels, may better distinguish between co-opted and angiogenic vessels since the latter are less mature and often lack pericyte coverage (16, 25). Double immunostaining using an EC marker and an antibody against Ki67, BrdU or proliferating cell nuclear antigen (PCNA) that detects proliferating cells may also aid in the detection of ongoing angiogenesis vs. co-opted endothelium (16).

To date, therapeutic strategies to combat pathological angiogenesis primarily rely on vascular endothelial growth factor (VEGF) signaling blockade. Despite initial optimism, the efficacy of anti-angiogenic pharmaco-monotherapies is typically short-lived, and drugs such as Bevacizumab (anti-VEGF-A monoclonal antibody that blocks ligand binding to VEGF-R2) have failed to deliver the promise of a cure for cancer. Improvements in patient survival are limited by acquired refractoriness and drug resistance (26–28). Despite these challenges, there are currently hundreds of clinical trials focusing on anti-VEGF treatment or some combination thereof. Anti-angiogenic strategies in cancer have been designed largely upon the premise that the tumor vasculature is composed of a normal, genetically stable population of endothelial cells. However, recent studies indicate that tumor endothelial cells (TEC) are more complex and dynamic than expected. Future clinical trial design would benefit from personalized application of anti-angiogenic therapies, likely in combination with checkpoint-inhibitor immunotherapy, based on a patient's specific tumor and TEC profile, to enable optimal responses (28).

In this review, we aim to illustrate how chemokine receptors support and serve as functional markers of the TEC population. We also discuss several studies that demonstrate the role of chemokine receptors within the heterogeneous TEC population and how these receptors may be relevant and appropriate targets for the treatment of cancer.

## Chemokine Receptor and Chemokine Structures and Roles in Cancer

Chemokine receptors maintain a classic seven transmembrane (7-TM) family structure. An important feature of 7-TM receptors is that they have a conserved disulfide bond between two cysteines, and part of what determines the large diversity of these families is how many amino acids separate those two cysteines (29). 7-TM proteins, including chemokine receptors, are notoriously challenging to target because (i) the high homology shared among closely related family members and in some cases their overlapping ligand-binding profiles make specificity an obstacle; (ii) proper physiologic conformation requires receptor expression in a plasma membrane (when synthesized separately, the extracellular domains rarely mimic endogenous conformation); and (iii) the epitope space available on the cell surface for chemokine receptors is highly limited (the amino-terminus and extracellular loops 1 and 2 are typically small in size, and chemokine binding occurs within a buried pocket lined by the 7-TM domains within the plasma membrane).

Chemokine receptors are well-known as leukocyte-expressed homing receptors that guide white blood cell localization throughout the body (30). Certain chemokine receptors are also overexpressed by cancer cells and tumor-associated stroma, the matrix that supports cancer cell proliferation and metastasis, and consists of infiltrating leukocytes, fibroblasts, pericytes, and endothelial cell populations. The role of chemokines and their receptors in the endothelium is particularly critical for tumor vascularization and metastatic spread.

Chemokines are classified into 4 categories (CC, CXC, XC, and CX_3_C) based on the position of conserved cysteine motifs (where X represents any non-cysteine residue). CXC chemokines are further divided into two groups with defined biological activities. The first set contains the ELR motif that induces chemotactic activity, inducing selectin-dependent leukocyte rolling on activated endothelium, followed by integrin mediated firm adhesion and transendothelial migration to inflamed sites (31–33). The second chemokine set lacks the ELR motif and does not induce chemotaxis across endothelial cells (34). CXCL12/SDF-1 is the only CXC chemokine that does not have the ELR motif, but is chemotactic and pro-angiogenic (35).

Tumor microenvironment-derived chemokines can induce vascular permeability and enable efficient tumor cell extravasation, promoting tumor cell colonization of distant sites (i.e., metastasis) (36). Tumor microenvironment-derived chemokines can induce endothelial cell recruitment by attracting the cells overexpressing their receptors, turning on feedback loops to induce angiogenic support for tumors. For example, CCL2, along with other CXC chemokines including CXCL1, CXCL2, CXCL3, CXCL5, CXCL6, CXCL7, and CXCL8 promote recruitment, migration, and proliferation of endothelial cells (37).

In general, upon binding to their ligands, chemokine receptors undergo conformational changes that allow the binding of G proteins to intracellular loop epitopes and the carboxy terminal tail of the receptors. This initiates a signaling cascade of activated second messengers that lead to cell motility and multiple other functional effects in the target cells. Major cellular processes are influenced significantly by chemokines via their receptors. Cancer cells can hijack the chemokine receptor system to enhance their survival and proliferation. Epithelial cells, stromal cells, and normal cells secrete chemokines that attract tumor cells overexpressing chemokine receptors and thus increase metastatic dissemination. Increased chemokine receptor expression can also support angiogenesis that feeds tumor growth and gives tumor cells additional survival signals or a survival advantage.

## Function of Known Chemokine Receptors Found in TEC

### Atypical Chemokine Receptors

#### ACKR1/DARC

Atypical chemokine receptor 1 (ACKR1), also known as Duffy antigen receptor for chemokines (DARC) binds more than 20 different chemokines, and is expressed in cerebellar neurons, venular ECs, and erythrocytes (38). ACKR1 is unique among vertebrate chemokine receptors because it can bind, with high affinity, both CC and CXC chemokines. ACKR1 is important for ligand transcytosis across endothelial layers, to enable presentation in the vascular lumen, as well as in buffering inflammatory chemokine levels in the circulation (39). DARC may serve as a marker to distinguish venular-ECs vs. non-venular-ECs (arterioles, capillaries) (40). In contrast to the other atypical receptors, ACKR1 is not believed to possess ligand-scavenging activity, but rather just present its ligands (39). Interaction of ACKR1 and tumor cell suppressor markers such as CD82, inhibit tumor cell proliferation and induce senescence by upregulating p21 and downregulating TBX2 (41, 42). ACKR1 may be involved in the regulation of angiogenesis, sequestering CXCL1, CXCL5, and CXCL8 (35, 43). In transgenic mice engineered to overexpress ACKR1 in endothelial cells (via a preproendothelin promoter/enhancer), ACKR1 decreased the pro-angiogenic properties of ELR^+^ CXC chemokines (44), whereas ACKR1 deficient mice showed increased levels of these chemokines as well as increased angiogenesis in a model of prostate adenocarcinoma (35).

#### ACKR3/CXCR7

Atypical chemokine receptor 3 (ACKR3), also known as CXCR7, has a modification in its DRYLAIV motif that does not allow it to bind heterotrimeric G-proteins after binding its ligands CXCL11 or CXCL12. Instead, it preferentially signals via the beta-arrestin pathway. ACKR3 is known to play a critical role in guiding progenitor cell migration during embryo- and organo-genesis (45). But following fetal development and birth, expression of ACKR3 protein is difficult to detect on the surface of cells or tissues, except in the context of cancer (46). ACKR3 is upregulated in many different cancer types including lung, cervical, pancreatic, myeloid, glial, and prostate cancer cells and brain cancer. ACKR3 may provide an advantage for tumor cells that favors their metastasis, driving cells through CXCL12 gradients by binding and degrading CXCL12–regulating bioavailability and gradient control, as it does during development. ACKR3 generally is not considered a chemotactic receptor, however, addition of CXCL12 enhances ACKR3+/CXCR4+ cancer cell migration across endothelial cells toward CCL19 and CXCL13, chemokines expressed by endothelial cells inside the lymph nodes (47, 48). Interestingly, this effect is abrogated when ACKR3 is inhibited with the small molecule CCX771, while not as diminished when CXCR4 is inhibited with AMD3100. Therefore, targeting ACKR3 could prevent lymph node entry and distant metastasis of CXCR4+/ACKR3+ positive tumor cells (48, 49). In addition, inhibiting ACKR3-dependent survival signals (50) may sensitize cells to chemotherapeutics or radiation (23). We and others have shown that ACKR3 is specifically up-regulated by activated (TNF-α treated) or inflamed endothelial cells while not in normal endothelial cells (29, 50–53). ACKR3 may also become upregulated in an unknown mechanism of tumor resistance to temozolomide (TMZ) where it could be identified as an independent factor for overall survival in the glioblastoma microenvironment (53), in line with mutations in isocitrate dehydrogenase mutations (IDH) and O6-methylguanine-DNA methyltransferase (MGMT) (54, 55). Moreover, inflammation augments ACKR3 expression on the abluminal surface of the brain microvessel endothelium, contributing to damage in the context of experimental autoimmune encephalomyelitis (EAE), and blocking ACKR3 decreases the damage to primary brain endothelial cells *in vitro* (56).

ACKR3 is upregulated in tumor endothelial cells, where it is induced under hypoxic and acidic conditions, distinctive features of the tumor microenvironment. CXCL12 secreted by TECs promotes ACKR3-mediated angiogenesis via ERK1/2 phosphorylation but not normal endothelial cells (NECs), indicating an autocrine/paracrine loop affects TEC proangiogenic properties (52). Therefore, ligand blocking inhibition of ACKR3 such as in the form of specific monoclonal antibodies that inhibit CXCL12 binding and beta-arrestin2 activation could significantly reduce TEC angiogenesis and decrease tumor burden acting as true and specific inhibitors of CXCL12 (29, 53, 57). VEGF stimulation upregulates ACKR3 expression in NECs (52). VEGF is stimulated by hypoxia and ACKR3 is stimulated by hypoxia inducible factor (HIF1a) (58). Thus, once the tumorigenesis process creates a hypoxic tumor microenvironment, ACKR3 expression by TEC may be induced by both HIF1a and VEGF.

### G-Protein-Coupled Chemokine Receptors

#### CXCR4

CXCR4 is the most common chemokine receptor overexpressed in human cancers and is implicated in over 25 different types of cancers (59, 60). CXCR4 is considered a novel marker in tumor endothelium, specifically on tip cells forming the sprouting tumor vessels within hepatocellular carcinoma (HCC). In the same study, high levels of TEC CXCR4 predicted poor prognosis for patients with HCC (61). Inflammatory cytokines derived from tumor conditioned monocytes/macrophages (Mo/Mϕ), especially TNF-α, upregulate CXCR4 expression on ECs (61). TNF-α induces activation of the Raf-ERK pathway and induces expression of CXCR4 on activated endothelial cells. CCR2 KO mice showed reduced infiltration of inflammatory Mo/Mϕ in tumor tissues and reduced vascular CXCR4 expression in HCC tumors (62). CXCR4 is among the genes enriched in tip cells vs. stalk endothelial cells, along with VEGFR2, platelet-derived growth factor (PDGF)- B, Dll4, and matrix metalloproteinase (MMP)14 (16, 28, 63).

CXCR4 is expressed by EPCs and is responsible for their maintenance in the bone marrow via CXCL12. VEGF/VEGF-R, which upregulates MMP9, and CXCL12/CXCR4 are considered the key pathways regulating bone marrow-EPC mobilization (64–66). On the other hand, inhibition of CXCR4 reduces VEGF secretion in tumor cells, which results in decreased neovascularization and tumor growth (67). CXCR4 is also expressed on the surface of LECs and is critical for lymphangiogenesis through CXCL12 stimulation (12, 68). Circulating levels of CXCL12 increase in patients who evade various anti-VEGF therapies, including rectal carcinoma with bevacizumab, GBM with cediranib, HCC with sunitinib, and soft tissue sarcoma with sorafenib (8, 69). The CXCL12/CXCR4 pathway is also involved in vessel co-option, vasculogenesis, fibrosis, lymphocyte trafficking, and cancer cell invasion, depending on the tumor and treatment. Clinical trials with AMD3100 (a CXCR4 small molecule antagonist) and the anti-VEGF mAb, bevacizumab are currently underway to treat recurrent GBM patients to address mechanisms of evasive resistance (8, 70).

#### CCR2

Although CCR2 is expressed in human endothelial cells, in the context of TEC, activation of CCR2 leads to phosphorylation of kinases JAK2 and p38MAPK and transcription factor Stat5, which enhances endothelial permeability and enables colon carcinoma extravasation and metastasis in preclinical mouse models (36). Pharmacological inhibition of CCR2 with a selective small molecule antagonist, CCX872, significantly suppressed tumor growth and enhanced survival in a spontaneous breast cancer model (*HER2/neu* transgenic mice) (71). A similar result was obtained using *HER2/neu* mice deficient in the single known CCR2 chemokine ligand, CCL2 (71). EPC express CCR2, migrate to CCL2 and contribute to tumor neovascularization (71). EPC development in the bone marrow and their mobilization to the blood was impaired in CCL2^−/−^ *HER2/neu* mice compared with WT *HER2/neu* mice, providing a possible mechanism for the observed anti-tumor phenotype (71). ChemoCentryx, Inc., recently reported the improved overall survival of CCX872-treated patients in an ongoing Phase 1b clinical trial for locally advanced/metastatic pancreatic cancer targeting CCR2 in circulating immune cell and myeloid derived suppressor cells (MDSCs). In addition, monoclonal antibodies that bind either CCL2 or CCR2 to inhibit the CCL2/CCR2 interactions (37, 72) have been tested in several inflammation-related diseases with varying but promising results (37).

#### CXCR2—The Receptor for Angiogenic Chemokines

CXCR2 is expressed on microvascular EC and binds all the ELR^+^ CXC-chemokines (CXCL1, CXCL2, CXCL3, CXCL5, CXCL6, CXCL7, and CXCL8) known to possess angiogenic activity and promote neovascularization (73, 74). In the gut, CXCR2 is expressed on human intestinal microvessels and on primary cultures of human intestinal microvascular endothelial cells (HIMEC) (75). CXCL8 secreted by malignant colon tumors had angiogenic effects on HIMEC (endothelial tube formation, EC chemotaxis), which was suppressed by CXCR2 antibody blockade or by ERK1/2 inhibition (75, 76). In lung cancer, CXCR2 is expressed on the vascular endothelium in human non-small cell lung carcinomas and in experimental mouse lung cancer models [orthotopic syngeneic Lewis lung carcinoma (LLC) tumors], and elevated levels of CXCL1, CXCL2/3 were detected in tumor vs. matched normal tissue controls (77). LLC tumor angiogenesis (as quantified by microvessel density) was inhibited in CXCR2-deficient mice or WT mice treated with anti-CXCR2 mAbs vs. controls, which correlated with increased necrotic area and reduced tumor growth *in vivo* (77). In a preclinical model of spontaneous prostate cancer, tumor growth was significantly suppressed in CXCR2-deficient mice compared with controls, with a corresponding reduction in tumor angiogenesis as measured by von Willebrand factor RNA expression (78). Similar results supporting a tumor-promoting angiogenic role for CXCR2 were also reported for pancreatic ductal adenocarcinoma (79) and ovarian cancer (80). Thus, targeting CXCR2, particularly in combination with immune checkpoint inhibitors, may offer new opportunities to slow or reverse cancer progression across multiple tumor types, as the approaches act via complementary anti-tumor pathways.

#### CXCR3—The Receptor for Angiogenesis Inhibiting Chemokines

CXCR3 interacts only with ELR^−^ angiostatic chemokines CXCL9, CXCL10, and CXCL11. Interferon (IFN) gamma-induces CXCL9/10/11 expression in endothelial cells, where the chemokines play an important role in the vascular recruitment of IFN-gamma-producing T cells to atherosclerotic plaques (81). CXCR3 is significantly overexpressed in vessels from primary kidney tumors compared to matched normal tissue vessels (82). CXCR3 expression on primary cultures of human microvascular endothelial cells (HMVECs) is limited to the S/G2-M phase of the cell cycle vessels (82). CXCL9, CXCL10, and CXCL11 block HMVEC proliferation (either spontaneous or basic fibroblast growth factor-induced) *in vitro*, which can be reversed by treatment with an anti-CXCR3 antibody (82). CXCR3 is a particularly complicated chemokine receptor due to its alternative splicing of the human gene, which generates 3 different isoforms, CXCR3A, CXCR3B, CXCR3alt, with CXCR3B mediating angiostatic activity on ECs (74). Differential expression of the three CXCR3 splice variants was reported in ovarian tumors, with overexpression of CXCR3alt and CXCR3A vs. normal tissue, while CXCR3B was downregulated vs. normal tissue, suggesting that a specific pattern of CXCR3 RNA transcript processing and expression favors tumor progression and metastasis (83). In alignment with this hypothesis, in a preclinical model of spontaneous prostate cancer, tumor growth was significantly increased in CXCR3-deficient mice compared with controls, with a corresponding increase in tumor angiogenesis as measured by von Willebrand factor RNA expression (78). Given the complexity of the splice variants and expression of CXCR3 on activated T cells, additional research is needed to determine if and how TEC-expressed CXCR3 may be targeted for cancer immunotherapeutic purposes.

## TEC-Targeted Cancer Therapy

### Advantages and Considerations

There are several advantages to targeting TEC to slow tumor progression. First, TEC are more accessible to systemically delivered agents than tumor cells in solid organ cancers. Second, because TEC are not typically immortal, they are less likely to develop resistance to therapies than neoplastic cells (3, 4). Third, Aird suggests that inhibition of a single TEC can suppress the growth and survival of up to 100 tumor cells (4), an exciting concept. Thus, treatment aimed toward the endothelium may have an amplifying anti-tumor effect. State of the art treatments such as tubulin-targeted drugs are the most advanced vascular disrupting agents (84). Combining anti-angiogenic and vascular disrupting agents that target the tumor vasculature may realize the full potential of vascular targeted therapies (84). Chemokine receptors are excellent targets for vascular disruption therapies because they are typically not expressed on cells that comprise vital organs, so adverse events may be limited. Another advantage is the potential for multimodal effects. For example, CXCR4-targeting would inhibit angiogenic signals and prevent *de novo* TEC expansion. Since existing TEC in many cases express CXCR4, targeting CXCR4 with a cytotoxic monoclonal antibody (mAb) could lead to tumor vascular disruption and neoplastic necrosis. CXCR4 is also present on anti-tumoral leukocytes and synergistically attracts leukocytes with other chemoattractants. Thus, the outcome of CXCR4 inhibition might differ depending on the tumor type or even individual patient. For example, since CXCR4 is upregulated on immune suppressive regulatory CD4+ T cells (85), targeting CXCR4 could diminish these immune checkpoint cells and potentially unleash a potent anti-tumor immune response.

### Recent Studies

Although most genes expressed in TEC are also upregulated in physiological angiogenic processes, there are important exceptions. The primary determinant of phenotypic heterogeneity in the context of the tumor is the surrounding physical microenvironment, which is typically hypoxic, acidic, and at a high interstitial fluid pressure (3, 86). Studies from high-throughput protein, gene arrays, and miRNA screens have identified unique molecular patterns in the tumor vasculature, but have failed to identify a consistency in TEC molecular signatures (3, 87). While the prevailing concept of the tumor endothelium was that of an admixture of cancer cells surrounded by normal endothelial cells, single cell expression profiling is currently demonstrating that tumor endothelium is distinct from the host's normal ECs.

A multicolor Cre-dependent marker system to trace clonality within the tumor endothelium showed that TECs and their vessels followed a pattern of dynamic clonal evolution. TECs were derived from a common precursor and evolved into a more invasive and immunologically silent phenotype. Gene expression profiling revealed selection for traits promoting upregulation of alternative angiogenic programs such as unregulated hepatocyte growth factor (HGF)/MET signaling and enhanced autocrine signaling through VEGF and platelet-derived growth factor (PDGF). As the TECs developed within the tumor, there was loss of normal EC function and markers including MAdCAM-1 that control lymphocyte homing. Changes in adhesive properties on tumor endothelial cells also showed decreased expression of lymphocyte-attracting chemokines CXCL16, CXCL13, CXCL12, CXCL9, CXCL10, and CCL19 (88). This study showed at high resolution how the tumor microenvironment co-opts endothelial cells and re-arranges their genetic program to drive tumor progression.

TECs and NECs isolated from human breast cancer tissues and reduction mammoplasty tissues were analyzed by single cell RNA sequencing (scRNA-seq) to characterize and compare their global gene expression profiles. Not surprisingly, chemokines and the GPCR pathways, which include the chemokine receptors, were some of the most differentially expressed genes and correlated with breast cancer (89). One limitation of the study is that the authors generate clusters from only 2 normal vs. 2 cancer endothelial cell sets, with a total of 280 viable ECs; thus, the conclusions are based on a relatively small data set.

In another study evaluating tumor endothelium by scRNA-seq analysis, the authors isolated TEC and NECs from xenografts of human colon carcinoma and successfully re-capitulated previously identified markers for tip and stalk cells. Most interesting was the effect of anti-angiogenic therapy on the TEC populations. The authors used either aflibercept (VEGF inhibitor), anti-Dll4 (Notch inhibitor), or a combination to treat the colon carcinoma xenograft model. Blockade of VEGF rapidly inhibited cell cycle genes and reduced the proportion of endothelial tip cells in tumors. In contrast, blockade of Dll4 promoted endothelial proliferation as well as tip cell markers while blockade of both pathways inhibited endothelial proliferation but preserved some tip cells (90). While the results are potentially exciting, these conclusions were made using TECs derived from contrived xenografted NSG tumor model mice, which have a limited capacity to model tumorigenesis in human cancer patients. Furthermore, the authors compared TEC to cardiac endothelial cells, which would presumably be quite different from anatomically matched NEC. In this same study, the authors identified additional potential tip-like cell markers such as Ramp3, Ednrb and Cldn5 as well as stalk-like cell markers such as Ackr1 and Tmem252 (90). These types of studies, at single cell resolution, give us the opportunity to appreciate the heterogeneity of the TEC population, confirm known markers, and allow us to propose previously unidentified roles for induced genes such as chemokine receptors, which can be challenging to identify specifically at the protein level due to lack of effective anti-mouse receptor antibodies.

## Perspective for the Future

Current technologies are allowing us to decipher at the single cell resolution the heterogeneity and functionality of the tumor endothelium. This will provide profound insight into mechanisms governing tumor leukocyte infiltration and functional activation via the quantitative analysis of adhesion molecule, chemoattractant, and costimulatory molecule expression. Single cell analysis of the endothelium may also help identify escape mechanisms from current anti-angiogenic and vascular disrupting agents and lead the way to effective countermeasures (88).

Understanding which chemokine receptors are responsible for the early development of the tumor EC but change as the TECs progress along with the rest of the tumor, will help us understand the dynamics of this heterogeneous population and identify appropriate targets for treatment. In the near future, we should seek to identify the characteristics of TECs at the single cell level to understand the transcriptional controls that are altered in NECs, EPCs, and/or cancer-stem-like cells that give rise to TECs in the tumor microenvironment. Understanding how to control this developmental switch will allow us to do what we cannot do today: control the position, abundance, and physical properties of tumor blood vessels. For example, in poorly-vascularized tumors, we may seek to increase the abundance and permeability of tumor vascular endothelium to allow better irrigation of a tumor with a drug or cellular treatment in a patient. In other cases, we may seek to optimally disrupt existing tumor-associated vascular endothelium by targeting key chemokine receptors for therapeutic purposes. As we enter the era of personalized medicine, sequence and expression analysis of neoplastic cells, and supporting TEC will guide the selection of optimal treatments. Given the role of TEC-expressed chemokine receptors in tumor progression, it is likely that chemokine receptor-targeted drugs will play a prominent role in most combination treatment strategies to treat cancer.

## Author Contributions

NS organized the research topic, wrote, and edited the manuscript. BZ wrote and edited the manuscript.

### Conflict of Interest Statement

The authors declare that the research was conducted in the absence of any commercial or financial relationships that could be construed as a potential conflict of interest.
